# A Transducing Bacteriophage Infecting Staphylococcus epidermidis Contributes to the Expansion of a Novel Siphovirus Genus and Implies the Genus Is Inappropriate for Phage Therapy

**DOI:** 10.1128/msphere.00524-22

**Published:** 2023-04-05

**Authors:** Taylor Andrews, J. Steen Hoyer, Karolyn Ficken, Paul D. Fey, Siobain Duffy, Jeffrey M. Boyd

**Affiliations:** a Department of Biochemistry and Microbiology, Rutgers University, New Brunswick, New Jersey, USA; b Department of Ecology, Evolution, and Natural Resources, Rutgers University, New Brunswick, New Jersey, USA; c University of Nebraska Medical Center, University of Nebraska, Omaha, Nebraska, USA; University of Michigan

**Keywords:** *Staphylococcus epidermidis*, bacteriophage genetics, bacteriophage therapy, extrachromosomal prophage

## Abstract

The effort to discover novel phages infecting Staphylococcus epidermidis contributes to both the development of phage therapy and the expansion of genome-based phage phylogeny. Here, we report the genome of an S. epidermidis-infecting phage, Lacachita, and compare its genome with those of five other phages with high sequence identity. These phages represent a novel siphovirus genus, which was recently reported in the literature. The published member of this group was favorably evaluated as a phage therapeutic agent, but Lacachita is capable of transducing antibiotic resistance and conferring phage resistance to transduced cells. Members of this genus may be maintained within their host as extrachromosomal plasmid prophages, through stable lysogeny or pseudolysogeny. Therefore, we conclude that Lacachita may be temperate and members of this novel genus are not suitable for phage therapy.

**IMPORTANCE** This project describes the discovery of a culturable bacteriophage infecting Staphylococcus epidermidis that is a member of a rapidly growing novel siphovirus genus. A member of this genus was recently characterized and proposed for phage therapy, as there are few phages currently available to treat S. epidermidis infections. Our data contradict this, as we show Lacachita is capable of moving DNA from one bacterium to another, and it may be capable of maintaining itself in a plasmid-like state in infected cells. These phages’ putative plasmid-like extrachromosomal state appears to be due to a simplified maintenance mechanism found in true plasmids of Staphylococcus and related hosts. We suggest Lacachita and other identified members of this novel genus are not suitable for phage therapy.

## INTRODUCTION

Staphylococcus epidermidis is a Gram-positive commensal bacterium of humans that is also an opportunistic pathogen: the most common source of nosocomial infections (1). S. epidermidis infections are becoming increasingly difficult to treat due to the prevalence of antibiotic-resistant strains (2). Instead of relying on continued development of new antibiotics (3), a promising alternative that is being approached with renewed interest is phage therapy, which uses bacteriophages to treat bacterial infections (4). Phage therapy utilizes the mechanism of lytic phage replication to kill infection-causing bacteria. While phages can be modified or selected under laboratory conditions to optimize their performance, phage therapy relies on the diversity of naturally occurring phages of pathogenic bacteria. New phages must be isolated from the environment, characterized, and assessed for therapeutic potential (5). However, phages that infect S. epidermidis remain largely undersampled and understudied, especially in comparison to its relative, Staphylococcus aureus (6).

In recent years, there has been increasing research and effort to isolate S. epidermidis phages (7–12). As part of this S. epidermidis phage prospecting, members of a novel genus were isolated in multiple parts of the world in 2021. Fanaei Pirlar et al. (13) isolated, characterized, and sequenced a double-stranded DNA (dsDNA) siphovirus, CUB-EPI_14 (GenBank accession no. ON325435.2), that is ~43 kb and has a narrow host range within S. epidermidis. Despite the genome of CUB-EPI_14 being labeled as likely temperate by PhageAI, which is normally disqualifying for a potential phage for therapy (14), CUB-EPI_14 lacks an integrase, and the authors suggest it could be a potential candidate for phage therapy (13).

Simultaneous with the work of Fanaei Pirlar et al., we also isolated a related phage from this novel genus by culturing wastewater on S. epidermidis, as did a third group, who has deposited their phage’s genome in GenBank without an accompanying paper (GenBank accession no. ON550478.1). We sequenced the genome of our representative (Lacachita) and conducted additional host range and transduction assays. We found three additional representatives of this novel genus in GenBank (including one from an S. epidermidis shotgun sequencing project) and analyzed these with the three cultured phage genomes. We identified in all genomes a common phage resistance gene and a partitioning protein that is associated with being maintained in a plasmid state. Combined with our observation that Lacachita can transduce antibiotic resistance genes, we propose that members of this novel genus are likely temperate and therefore inappropriate for phage therapy.

## RESULTS

### Isolation of Lacachita.

Phages capable of forming plaques on S. epidermidis 1457 were successfully isolated from samples of wastewater influent from a treatment plant in the mid-Atlantic United States. Concentrated samples of the unenriched wastewater did produce plaques on S. epidermidis 1457, but the enriched wastewater produced orders of magnitude more plaques. Plating the host alone (without wastewater) did not produce any plaques. During the isolation procedure, a total of 11 plaques were harvested for potential further work. Of these isolated plaques, two were chosen for sequencing. Upon sequencing and assembly of the genomes, it was discovered that the two were 100% identical, and so only one (Lacachita) was further characterized.

### Lacachita genome and annotation.

Lacachita has a 46,473-bp dsDNA genome that is likely circular (GenBank accession no. OP142323). It contains 72 predicted open reading frames (ORFs), 19 putative promoters, 1 putative noncoding RNA (which encodes a group I catalytic intron), 19 putative rho-independent terminators, and no predicted tRNAs. Several similar phage genomes were identified by BLAST (>95% identity over ≥93% of the genome), and the Lacachita genome was linearized and oriented to mimic the genomes of its close relatives, which were also isolated on S. epidermidis (accession no. ON550478.1 and ON325435.2) (Table 1).

**TABLE 1:** Other proposed members of the novel genus

Phage genome	Accession no.	Sample type	Isolation location	Length (bp)	% query coverage of Lacachita	% identity to Lacachita	Phage AI predicted lifestyle
Staphylococcus phage Sazerac, complete genome	ON550478.1	Cultured isolate	IL, USA	46,428	94	96.27	Temperate
Staphylococcus phage CUB-EPI_14, complete genome	ON325435.2	Cultured isolate	Germany	46,098	93	96.19	Temperate
Uncultured Caudovirales phage clone 9S_3	MF417888.1	Uncultured isolate	South Africa	45,052	93	95.53	Temperate
TPA *Myoviridae* sp. isolate ct5pN1	BK030923.1	Metagenome-assembled genome	USA	46,472	95	98.64	Temperate
Contig of Staphylococcus epidermidis isolate Sep_B35_CVC_2019, whole-genome shotgun sequence (proposed prophage)	NZ_CAJUVG010000006	Whole-genome shotgun sequence from S. epidermidis isolate	Portugal	46,658	98	96.47	Temperate

**FIG 1:**
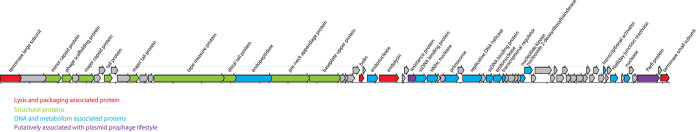
Lacachita genomic map. ORFs are annotated with predicted protein products.

Lacachita’s putative protein products contain an expected assortment of phage proteins and some hypothetical proteins (Fig. 1). Nine structural proteins were identified, which were similar, by blastp, to those of siphoviruses with long, noncontractile tails. Lacachita has both a holin and an endolysin, and 14 proteins involved in DNA replication and metabolism were identified. Two ORFs are associated with a plasmid prophage lifestyle: a ParB-like protein and a potential phage resistance protein. The remaining 43 ORFs in the Lacachita genome are either hypothetical (34 ORFs) or are identified only with a protein family or as including a known domain (9 ORFs).

### Lacachita is part of novel genus, along with other proposed members.

Four other phage genomes were identified by blastn to be relatives of Lacachita, and another was identified by blastp (Table 1). Of these five relatives, only CUB-EPI_14 (accession no. ON325435.2) has yet been thoroughly described (13). The authors of that article noted that CUB-EPI_14 appears to represent a novel genus and identified two other potential members of the genus via calculation of intergenomic distance—Caudovirales phage clone 9S_3 (accession no. MF417888.1) and third party annotation (TPA) *Myoviridae* sp. isolate ct5pN1 (accession no. BK030923.1). These two phages were also independently identified as relatives of Lacachita during our searches, and so our analysis complements and bolsters the evidence for these phages representing a new genus. The genome of another cultured phage, Sazerac (accession no. ON550478.1), was deposited in GenBank after the manuscript about CUB-EPI_14 was submitted for publication, and we propose that Sazerac is also part of this novel genus. The final relative, Sep_B35_CVC_2019 (accession no. NZ_CAJUVG010000006), was identified due to its consistent protein sequence identity to Lacachita protein products. Although Sep_B35 is catalogued in NCBI as a contig of a S. epidermidis whole-genome shotgun sequence, we argue that this contig represents a full phage genome from an infected S. epidermidis strain. Furthermore, since the sample of S. epidermidis was sequenced as a bacterial shotgun sequencing project, not labeled as a study in phage infection, we suggest that the Sep_B35 genome represents a prophage that was being maintained within the S. epidermidis isolate at the time it was sequenced.

**FIG 2:**
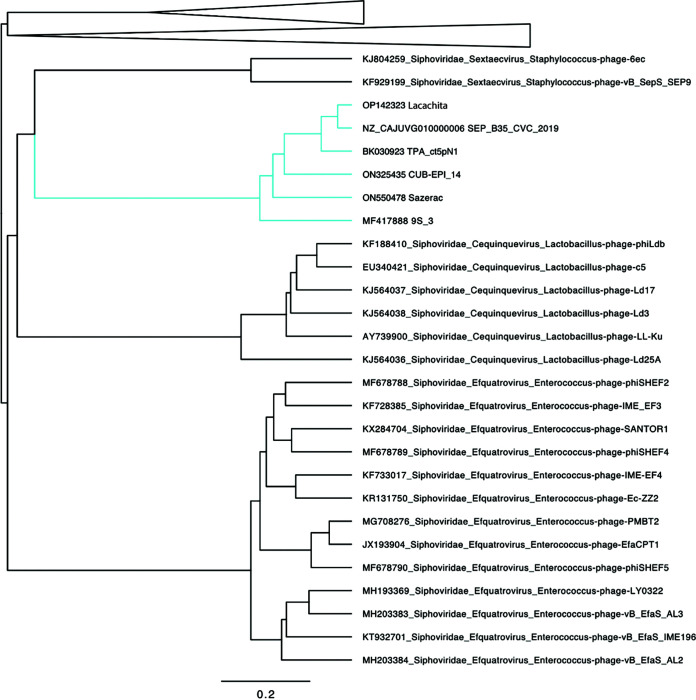
Phylogenetic tree of dsDNA prokaryotic viruses from GRAViTy, collapsed to focus on Lacachita and its relatives. Labels include GenBank accession numbers, family, order, and genus assignments and phage names. The six genomes comprising the novel genus, including Lacachita, are in the blue-green clade.

**FIG 3:**
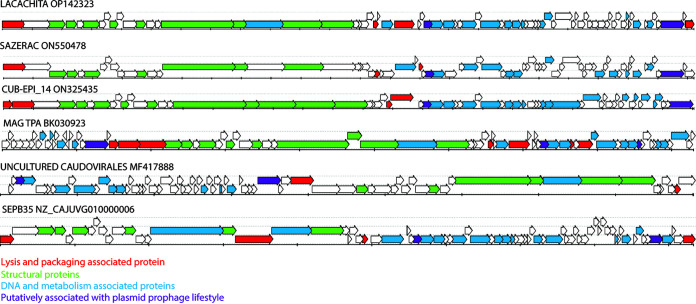
Genomic maps of close relatives of Lacachita. ORFs are color coded according to the categories of predicted protein products described in GenBank accession numbers. Genomes are presented using the coordinate numbering system from their GenBank entries. The genomes of MF417888 and NZ_CAJUVG010000006 were reversed to match the orientation of Lacachita.

Taxonomic assignment of Lacachita and its relatives confirmed that these phages represent a novel genus within the family *Siphoviridae* (Fig. 2). The six genomes form a monophyletic clade, clustered near the *Sextaecvirus* infecting other staphylococci, among other siphoviruses. There was strong support for this group forming a novel genus (symmetrical Theil’s uncertainty correlation of 0.863). Genomic maps of the six members of the putative genus reveal some observable regions of synteny (Fig. 3).

Following the example of Fanaei Pirlar et al. (13), we ran the six genomes through the PhageAI lifestyle classifier. We found that Lacachita and all members of this putative novel genus were predicted to be temperate with at least 99.95% confidence (Table 1). Three of the five proposed relatives of Lacachita are uncultured, putative phages. Based on the length and query coverage of these genomes compared to those of cultured isolates, it is likely that these represent essentially complete genomes.

### Host range results.

Of the 7 S. epidermidis strains tested in this project, 5 μL of Lacachita lysate was found to be capable of lysing S. epidermidis strains 1457, NRS101 (RP62a), B72-22, 158-22, and B138-22. Of the other Staphylococcus species strains tested, 5 μL of Lacachita lysate was found to be capable of lysing Staphylococcus capitis B65-22 and B1931-21 and Staphylococcus lugdunensis B50-22 (Table 2).

**TABLE 2:** Lacachita host range as determined by 5-μL spots of serial dilutions of lysates

Species	Strain	Highest dilution of Lacachita lysate that produced lysis
*S. epidermidis*	1457	10^−5^
	158-22	Lysate
	B138-22	Lysate
	B72-22	Lysate
	B76-22	
	B64-22	
	NRS101(RP62a)	10^−2^
	ATCC 12228	
*S. capitis*	B65-22	10^−2^
	B1931-21	10^−2^
*S. lungenensis*	B50-22	Lysate
*S. haemolyticus*	B1869-21	
	157-22	
*S. hominis*	160-22	
	B124-22	
*S. simulans*	B149-22	
	B1781-21	
*S. aureus*	B21-11	
	SH1000	
	MW2	
	N315	
*S. warneri*	B21-22	

### Lacachita is capable of transduction and lysogeny.

Transduction assays were conducted three separate times, and in 5/6 cases, Lacachita was capable of transducing plasmid-encoded erythromycin resistance to erythromycin-sensitive S. epidermidis 1457.

Spotting of serial dilutions of Lacachita lysate on bacterial lawns of transduced S. epidermidis 1457 revealed that these transduced cells were 100-fold less susceptible to lysis than S. epidermidis 1457 that had not undergone transduction. While the highest dilution of the lysate able to produce lysis on S. epidermidis 1457 was 10^−6^, the highest dilution of the lysate able to lyse transduced S. epidermidis 1457 was 10^−4^.

**FIG 4:**
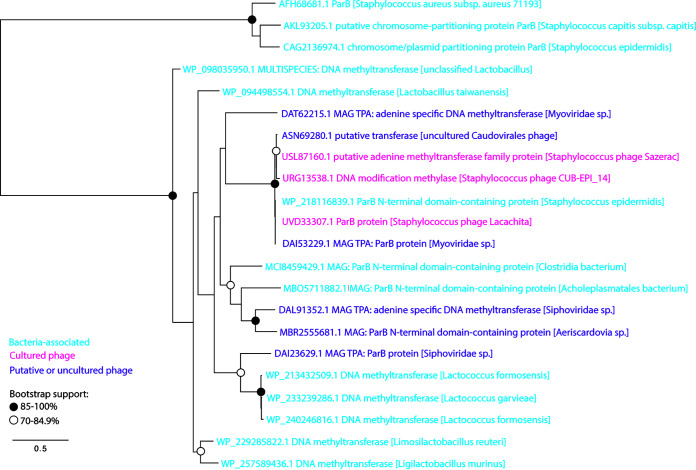
Maximum likelihood tree of protein sequences similar to Lacachita’s ParB protein (UVD33307.1) and identified ParB proteins from Staphylococcus genomes. Protein names are color coded according to their source organism. Bacterium-associated proteins are shown in blue, cultured phage proteins are shown in pink, and putative or uncultured phage proteins are shown in purple. Clades with moderate or strong bootstrap support are shown with open (70 to 84.9%) or closed (85 to 100%) circles.

We were unable to identify a putative integrase gene in the genomes of Lacachita or its close relatives. ORF analysis of Lacachita and its close relatives revealed the presence of putative *parB* and common phage resistance gene (Fig. 1) in all six genomes (Table 3), which is partial evidence that these phages are temperate and suggests the prophages are maintained extrachromosomally. We elected to classify Lacachita ORF70 (UVD33307.1) as “ParB protein” because the highest blastp result (99.8% identity) was annotated as a ParB protein (MAG TPA ParB protein [*Myoviridae* sp.] accession no. DAI53229.1). We conducted a phylogenetic analysis of the ParB-like proteins from this putative genus and related sequences (identified by blastp) and annotated ParB proteins from Staphylococcus genomes (Fig. 4). The sequences from Lacachita’s putative genus form a robust clade (99.7% bootstrap support), and several of the more distantly related sequences from other phage genomes are also classified as ParB proteins. The sister group (accession no. DAT62215.1) shares 51.2% identity and 99% query coverage with Lacachita’s ParB protein. While some bacterial ParB proteins were identified by the blastp search, they were not from Staphylococcus: the Staphylococcus ParB sequences formed an outgroup to the sequences identified by blastp. Our decision to classify this ORF as *parB* was bolstered by the efficiency of plating results on transduced S. epidermidis cells; Lacachita appears to be temperate. We elected to classify Lacachita ORF30 (UVD33267.1) as a “resistance protein” because its highest NCBI blastp result (100% identity) was the “resistance protein” of accession no. DAI53234.1: MAG TPA resistance protein (*Myoviridae* sp.). The other blastp hits (within and outside this putative genus) are from phage and Staphylococcus proteins with Siphovirus-gp157 protein family annotations (pfam05565), members of which have been experimentally shown to confer phage resistance in streptococci (15, 16).

**TABLE 3:** Protein comparison by phage genome

Phage genome	Original annotation	Accession	% query coverage compared to Lacachita	% identity to Lacachita
ParB comparison				
Sazerac	Putative adenine methyltransferase family protein	USL87160.1	100	98.20
CUB-EPI_14	DNA modification methylase	URG13538.1	100	95.99
Uncultured Caudovirales phage clone 9S_3	Putative transferase	ASN69280.1	90	97.79
TPA *Myoviridae* sp. isolate ct5pN1	ParB protein	DAI53229.1	100	99.80
Sep_B35_CVC_2019 proposed prophage	ParB N-terminal domain-containing protein	WP_218116839.1	52	100
Phage resistance protein comparison				
Sazerac	Hypothetical protein	USL87122.1	100	96.89
CUB-EPI_14	Hypothetical protein	URG13503.1	100	83.23
Uncultured Caudovirales phage clone 9S_3	Hypothetical protein	ASN69320.1	100	88.82
TPA *Myoviridae* sp. isolate ct5pN1	Resistance protein	DAI53234.1	100	100
Sep_B35_CVC_2019 proposed prophage	Siphovirus Gp157 family protein	WP_218116871.1	100	83.23

## DISCUSSION

### Lacachita is part of a novel genus.

Our GRAViTy analysis strongly suggests that Lacachita and its relatives belong to a siphovirus genus recently described by Fanaei Pirlar et al. (13), who characterized CUB-EPI_14 as belonging to a novel genus along with 9S_3 and ct5pN1. We have also expanded this putative genus by two more phages: Sazerac and the Sep_B35_CVC_2019 putative prophage. Based on the high genetic identity between CUB-EPI_14 and the other genomes, we can assume all members of this genus have long, noncontractile tails (13). Members of this genus have already been found on three continents, and we anticipate further isolates will be characterized in the upcoming years. Additional hosts may be identified for these phages as well, as we have expanded the potential hosts for members of this genus to include other Staphylococcus species than S. epidermidis (relative to Fanaei Pirlar et al. [13]).

### Lacachita and its relatives are not suitable for phage therapy.

However, unlike Fanaei Pirlar et al. (13), we do not think members of this genus should be used for phage therapy. There are several characteristics that are typically screened for when assessing whether a phage could be used for phage therapy, including host range, phage virulence, transduction potential, stability against environmental pressures, and the presence of toxin genes (17). Bioinformatic analysis suggests members of this genus are temperate, which is contraindicated for phage therapy. Temperate phages capable of transduction have the potential to increase the pathogenicity of lysogenized bacteria by carrying virulence factors between hosts (18). This is observed in temperate phages of S. epidermidis that can mobilize antibiotic resistance plasmids (19). In the interest of self-preservation, prophages also typically cause lysogenized bacteria to become immune to lytic infection by other phages that share similar repression systems (17). None of the members of this putative genus has an integrase gene, which is a key indicator of a temperate lifestyle because it allows stable integration of the phage genome into that of its host (14). Instead, the signal that PhageAI is picking up on in the phage genomes may be the presence of the *parB* gene (typically found as a *parA*-*parB* pair, implying a ParAB-*parS* system for chromosome segregation [20]) and the putative phage resistance gene, which is not required in phages that only rapidly lyse their host cells (21). Some temperate phages are known to be maintained in their bacterial host cells as extrachromosomal circular plasmids and maintain their presence in their hosts with similar mechanisms to plasmids (21–23). Some phage prospecting projects anticipate that these plasmid-like prophages may be isolated (24) but require that genomes have both *parA* and *parB* partitioning protein ORFs identified to be considered temperate (https://seaphages.org/forums/topic/4367/). To our knowledge, there are no characterized phages, temperate or otherwise, that contain only a gene for ParB, which binds to specific DNA sequences (*parS* sequences, which vary among bacteria [25]). A blastp search with the ParB of Lacachita found only members of its genus and 50% coverage to other phage proteins (typically the N terminus of ParB [data not shown]). Nonetheless, we did not find a ParA homolog, which is an ATPase that assists with localization of ParB (26, 27) in these six phage genomes. However, members of the genera *Staphylococcus* and *Streptococcus* are known to use only a ParB-*parS* system to ensure chromosome segregation without ParA (25); Staphylococcus aureus plasmid SK1 has a ParB-*parS* system as well (28). Therefore, extrachromosomally maintained prophages of these hosts may also not need ParA in order to stably vertically transmit to daughter cells.

There is no perfect test for whether a phage is temperate and capable of creating a lysogen. We observed intermittent turbidity of plaques on S. epidermidis 1457 and in the spot plating experiments on other Staphylococcus strains, which is often considered an important phenotype of temperate phages (21). However, turbidity can be affected by many factors (21). Importantly, we have repeated evidence of Lacachita’s capacity to transduce erythromycin resistance to a previously susceptible strain of S. epidermidis. Transduction is a phenomenon typically associated with temperate phage, although it can be due to “pseudolysogeny,” or the formation of a carrier state (29–31). Our experimental evidence of Lacachita’s ability to confer phage resistance to transduced cells is more indicative of lysogeny. Many temperate phages do not confer complete resistance to infection with that phage, and a 100-fold reduction in efficiency of plating is consistent with the behavior of other temperate phages (32, 33). Regardless of the durability of lysogeny with Lacachita, any transduction ability is empirical evidence that Lacachita and its close relatives should not be used in phage therapy.

Phage therapy remains a promising avenue of research for treating S. epidermidis infections, but members of this genus are not appropriate therapeutic agents. Genetic engineering is one way to modify temperate phages to be more appropriate therapeutic candidates (34), but that approach is controversial (35). Additional isolation of S. epidermidis phages is needed to find obligately lytic phage.

## MATERIALS AND METHODS

### Wastewater sample screening.

Aliquots of wastewater influent from a treatment plant in the mid-Atlantic United States were obtained in March 2021 and were screened for lytic phages effective against S. epidermidis 1457 (36). Five milliliters of the wastewater samples was combined with 0.15 g powdered tryptic soy broth (TSB) medium, 25 μL of 1 M CaCl_2_, and 50 μL bacterial broth culture (S. epidermidis 1457) and then incubated overnight at 37°C. A second sample of wastewater underwent the same procedure without the addition of host bacteria. After incubation, the mixtures were centrifuged at 3,220 × *g* for 15 min and the supernatant was passed through a 0.22-μm-pore filter. One hundred microliters of the filtrate was cultured with 100 μL of 10^−1^ bacterial dilution of an overnight culture using the pour plate method (in 3 mL of 0.3% molten tryptic soy agar [TSA] combined with 25 μL of 1 M CaCl_2_, vortexed, and poured onto TSB–1% agar plates). The plates were then incubated overnight at 37°C and examined for the presence of plaques.

### Phage isolation.

In order to purify phages identified during the screening process, isolated plaques were picked up using a sterile glass pipette tip, and the agar was deposited into a culture tube containing 2 mL TSB, 50 μL bacterial broth culture, and 25 μL of 1 M CaCl_2_ and incubated overnight at 37°C. This liquid culture was then centrifuged at 3,220 × *g* for 15 min, and the supernatant was passed through a 0.22-μm-pore filter. The filtrate was diluted, and 100 μL of this diluted filtered supernatant was combined with 100 μL 10^−1^ bacterial dilution, 25 μL of 1 M CaCl_2_, and 3 mL of molten TSA, vortexed, and poured onto TSA plates. The plates were then incubated overnight at 37°C and examined for plaques. This subculturing procedure was performed a total of three times to yield a purified, enriched phage stock.

### DNA isolation and sequencing.

The DNA genome of one isolated phage was extracted using a Qiagen QIAamp MinElute virus spin kit. Paired-end Illumina sequencing was performed at MiGS (Microbial Genome Sequencing Center [now SeqCenter]). Reads were analyzed using CPT Galaxy Phage genome assembler v.2021.01 Workflow (37), which uses SPAdes Galaxy v.3.12.0 (38). This yielded three assembled contigs. These contigs were aligned manually using Aliview (39) to verify that they were identical (except for short regions of duplication due to the likely circular genomes having been assembled linearly) and to produce a complete genome without such duplications. The Lacachita genome was reoriented to mimic the linearization of relatives found using NCBI Standard Nucleotide BLAST.

### Genome annotation.

The Lacachita genome was annotated using Prokka (v.1.14.6; Galaxy) with the parameters Kingdom: Viruses (40). Predicted ORFs were annotated further using NCBI Standard Protein BLAST, and the sequences producing significant alignments were analyzed to determine functional gene annotations for Lacachita. When the BLAST search produced multiple identical hits, we chose the annotation that was most relevant to a phage lifestyle (e.g., the name given in another phage genome). Phylogenetic analysis of the Lacachita genome and other dsDNA phage genomes was performed with GRAViTy v.1.1.0 (Genome Relationships Applied to Virus Taxonomy; http://gravity.cvr.gla.ac.uk/) (41).

Further functional annotation was performed. Promoter sequences were predicted by inputting the Lacachita genome into the Genome2D Prokaryote Promoter Prediction tool (42). Rho-independent termination sites were predicted using the ARNold web tool (43). Noncoding RNAs were found using Rfam (44). TRNAscan-SE was used to search the Lacachita genome for transfer RNAs (45).

### Comparison to related phage genomes.

To identify close relatives of Lacachita, the assembled Lacachita genome was used to query NCBI Standard Nucleotide BLAST. Other phage relatives were identified by searching predicted Lacachita ORFs using NCBI Standard Protein BLAST and making note of organisms with consistent protein homology to Lacachita ORFs, whose genomes were then compared to Lacachita directly using NCBI Align Sequences Nucleotide BLAST. Lacachita and identified relatives were analyzed with GRAViTy v.1.1.0 to determine their taxonomy (41). GRAViTy results were visualized using FigTree v.1.4.4 (http://tree.bio.ed.ac.uk/software/figtree/). The protein products of Lacachita predicted ORFs were analyzed to identify potential indicators of phage lifestyle, and the genomes of Lacachita and its relatives were also analyzed using the PhageAI lifestyle classifier algorithm (46).

### ParB protein maximum likelihood tree.

Two sets of ParB-like protein sequences were collected for phylogenetic analysis: Lacachita ParB blastp hits and annotated ParB sequences from Staphylococcus genomes. Sequences were aligned by MUSCLE (47), and the alignment was checked by eye. The aligned sequences were used to build a maximum likelihood tree with PhyML (48) on the Montpellier Bioinformatics Platform (http://www.atgc-montpellier.fr). The LG substitution model with empirical amino acid frequencies and estimated proportion of invariant sites were used, and 1,000 bootstrap replicates were run.

### Host range.

The host range of Lacachita was explored via spot plating on multiple strains of S. epidermidis and several other Staphylococcus species isolates. The S. epidermidis strains tested were 1457, 158-22, B138-22, B72-22, B76-22, B64-22, NRS101 (RP62a), and ATCC 12228. We tested other Staphylococcus species isolates: S. hominis (160-22, B124-22), *S. haemolyticus* (B1869-21, 157-22), *S. simulans* (B149-22, B1781-21), *S. capitis* (B65-22, B1931-21), *S. lugdunensis* (B50-22), *S. warneri* (B21-22), and S. aureus (LAC WT, SH1000, MW2, N315). With the exception of S. epidermidis strains 1457, ATCC 12228, and NRS101 (RP62a) and the S. aureus strains, the isolates are deidentified clinical isolates that were collected at the University of Nebraska Medical Center Clinical Microbiology Laboratory. Pour plates of each strain were prepared by combining 3 mL of 0.7% molten TSA, 25 μL of 1 M CaCl_2_, and 10 μL bacterial overnight culture, vortexing the mixture, and pouring it onto TSA–1% agar plates. Once the top agar solidified, 5 μL of a dilution series (1 to 10^−9^) of high-titer Lacachita lysate was spotted onto the surface. As a control, 5 μL of TSB was also spotted onto the plates. The plates were then incubated overnight at 37 °C and examined for evidence of lysis. Experiments were conducted in triplicate.

### Transduction.

In order to determine whether Lacachita possesses transduction abilities, a plasmid transduction experiment was performed. A modified S. epidermidis strain (1457 *saeR*/pNF155) carrying a 9-kb plasmid that is marked with an erythromycin resistance gene served as a donor strain, and erythromycin-sensitive S. epidermidis 1457 served as a recipient strain (49). Phage-bacterial cocultures were prepared with 2 mL of S. epidermidis 1457 *saeR*/pNF155 overnight culture (grown in TSB with 10 μg/mL erythromycin), which was combined with 5 mL TSB, 100 μL 1 M CaCl_2_, and 100 μL Lacachita purified phage stock. These bacterial-phage cocultures were incubated overnight at 37°C. The following day, Lacachita phages were harvested from the cocultures by centrifugation (13,000 × *g* for 3 min) and the resulting supernatant was then filtered using sterile 0.22-μm-pore filters to remove bacterial cells. This filtered supernatant was then combined with overnight cultures of S. epidermidis 1457 recipient strain: 500 μL S. epidermidis 1457 culture, 500 μL TSB, 100 μL 1 M CaCl_2_, and 100 μL of the harvested Lacachita donor phage preparation. These cocultures were incubated at 37°C for 1 h. Following incubation, 400 μL of 1 M sodium citrate was added to each, each tube was vortexed to mix, and each coculture was transferred to a microcentrifuge tube. Cells were pelleted by centrifugation at 13,000 × *g* for 2 min, and the supernatant was discarded. The cells were resuspended in 1 mL TSB and centrifuged again at 13,000 × *g* for 2 min. The cells were then resuspended in 200 μL TSB and plated on TSA plates containing 10 μg/mL erythromycin and 2 mM sodium citrate. As negative controls, erythromycin-sensitive S. epidermidis strain 1457 was plated on erythromycin-containing TSA plates and the Lacachita phage stock was spotted onto erythromycin-containing TSA plates. Inoculated plates were incubated overnight at 37°C and were then examined for the presence of bacterial growth. The transduction experiments were performed six times.

### Assessment of phage resistance of lysogenized bacterial cells.

To determine whether transduced bacteria were resistant to new lytic Lacachita infection, we spotted serial dilutions of Lacachita lysate onto lawns of transduced S. epidermidis 1457 cells. We obtained transduced cells using a modification of the transduction protocol described above (excluding sodium citrate from the agar plates), and representative resulting colonies were streaked onto erythromycin-containing TSA plates. Liquid cultures were inoculated with isolated colonies in 10 mL TSB containing 10 μg/mL erythromycin and were incubated overnight at 37°C. Pour plates of lysogenized cells were prepared by combining 3 mL of 0.7% molten TSA, 25 μL of 1 M CaCl_2_, 10 μL bacterial overnight culture, and 3 μL 10 μg/mL erythromycin, vortexing the mixture, and pouring it onto TSA plates. Once the top agar solidified, 5 μL of a dilution series (from 1 to 10^−9^) of high-titer Lacachita lysates was spotted onto the surface. As a control, 5 μL of TSB was also spotted onto the plates. For comparison, this was also performed on S. epidermidis 1457 that had not undergone transduction and potential lysogeny. The plates were then incubated overnight at 37°C and examined for evidence of lysis. Experiments were conducted in triplicate.
